# How the Competition for Cysteine May Promote Infection of SARS-CoV-2 by Triggering Oxidative Stress

**DOI:** 10.3390/antiox12020483

**Published:** 2023-02-14

**Authors:** Annamaria Vernone, Loredana Bergandi, Simone Pernice, Gianpiero Pescarmona, Francesca Silvagno

**Affiliations:** 1 Department of Neurosciences “Rita Levi Montalcini”, University of Torino, Via Cherasco 15, 10126 Torino, Italy; 2 Department of Oncology, University of Torino, Via Santena 5 bis, 10126 Torino, Italy; 3 Department of Computer Science, University of Torino, Via Pessinetto 12, 10149 Torino, Italy

**Keywords:** SARS-CoV-2, SARS-CoV-2 receptor, ACE2, ROS, cysteine, glutathione, spike, amino acid availability

## Abstract

SARS-CoV-2 induces a broad range of clinical manifestations. Besides the main receptor, ACE2, other putative receptors and co-receptors have been described and could become genuinely relevant to explain the different tropism manifested by new variants. In this study, we propose a biochemical model envisaging the competition for cysteine as a key mechanism promoting the infection and the selection of host receptors. The SARS-CoV-2 infection produces ROS and triggers a massive biosynthesis of proteins rich in cysteine; if this amino acid becomes limiting, glutathione levels are depleted and cannot control oxidative stress. Hence, infection succeeds. A receptor should be recognized as a marker of suitable intracellular conditions, namely the full availability of amino acids except for low cysteine. First, we carried out a comparative investigation of SARS-CoV-2 proteins and human ACE2. Then, using hierarchical cluster protein analysis, we searched for similarities between all human proteins and spike produced by the latest variant, Omicron BA.1. We found 32 human proteins very close to spike in terms of amino acid content. Most of these potential SARS-CoV-2 receptors have less cysteine than spike. We suggest that these proteins could signal an intracellular shortage of cysteine, predicting a burst of oxidative stress when used as viral entry mediators.

## 1. Introduction

The seventh human-infecting coronavirus (HCoV), the severe acute respiratory syndrome virus 2 (SARS-CoV-2), is a positive-sense RNA virus with a large single-stranded RNA genome of approximately 30 kb in length [1]. The SARS-CoV-2 genome contains 14 open reading frames (ORFs), preceded by transcriptional regulatory sequences (TRSs) [2]. The genome encodes three classes of proteins. Two large polyproteins, the polyprotein 1a (pp1a or R1A) and the polyprotein 1ab (pp1ab or R1AB), are subsequently processed by two cysteine proteases into 16 non-structural proteins (NSP1-16) which form the complex replicase machinery. At the 3′ end, the viral genome codes four major structural proteins (spike, envelope, membrane, and nucleocapsid proteins), which are components of the mature virus playing a crucial role in viral structure assembly and integrity or, as in the case of the spike protein, for viral entry into the host [2,3].

The infection is started by the interaction between the viral envelope and a broad range of surface molecules of the host cell, followed by the recruitment of proteases that activate spike protein and allow the interaction with the receptor angiotensin-converting enzyme 2 (ACE2). Two cellular proteolytic systems are hijacked by SARS-CoV-2 to ensure the adequate processing of its spike protein. In the priming step, furin divides the protein into two subunits, S1 and S2, held together by noncovalent interactions [4,5]. The S1 ectodomain undergoes a conformational change that exposes its receptor-binding domain (RBD), which recognizes the widely expressed receptor ACE2. The cleavage at S2′ site by the cell surface type II transmembrane serine protease 2 (TMPRSS2) triggers a broad protein rearrangement [6], leading to the separation of the S1 and S2 subunits and the exposure of the hydrophobic α-helical fusion peptide (FP), thus favoring fusion of viral and host cell membranes necessary for viral entry, followed by the release of viral RNA into the host cytoplasm [5]. In the cytoplasm, viral RNA utilizes the host and its own machinery to replicate its genetic material and assemble new viral particles [5].

The role of host cell proteases in SARS-CoV infection is not limited to cleavage of the spike protein; in fact, spike binding to ACE2 activates the disintegrin and metallopeptidase domain 17/tumor necrosis factor-converting enzyme (ADAM17/TACE) and its close relative ADAM10 [7], which cleave the ectodomain of ACE2, resulting in shedding of ACE2 [8]. This processing seems to play an important role in SARS-CoV entry and pathogenesis [9]. Recently, other host receptors and/or co-receptors that promote the entry of SARS-CoV-2 into cells have been reported [10,11,12,13,14,15,16,17,18,19]; the variety in the use of the receptors may provide an explanation for the high infectivity of this coronavirus.

The surface expression of ACE2 is reduced in SARS-CoV infections due to the spike protein internalization followed by ACE2 lysosomal degradation [20] and because the shed soluble form of ACE2 (sACE2) lacks the membrane anchor and circulates in small amounts in the blood [21]. Normally, the angiotensin-(1-7) peptide synthesized by ACE2 activity causes vasodilatation, angiogenesis, and anti-inflammatory, anti-oxidative, and anti-apoptotic effects, which prevent or attenuate the cellular damage induced by oxidative stress. In coronavirus infection, the result of the downregulation of ACE2 is the toxic overaccumulation of the angiotensin II (Ang-II), the product of the opposite angiotensin-converting enzyme (ACE) activity, which increases the production of reactive oxygen species (ROS) through the activation of NADPH oxidase and the generation of peroxynitrite anions. Thus, the exacerbated inflammation leads to acute respiratory distress syndrome and fulminant myocarditis in coronavirus disease 2019 (COVID-19) [22]. A different balance of ACE/ACE2 and the variable ratio of ROS over antioxidant defenses can explain the heterogeneous responses to infection, the severity of symptoms, and the extended recovery times caused by the same virus [23].

The main antioxidant protective molecule abundantly produced by every tissue is glutathione (GSH), a tripeptide composed of glutamate, cysteine, and glycine, whose synthesis is induced by oxidative stress and is potentiated by vitamin D [22]. The biosynthetic pathway of GSH consists of two ATP-dependent reactions catalyzed by glutamate-cysteine ligase (GCL) and glutathione synthase (GS) and requires full availability of the two limiting amino acids cysteine (Cys) and glycine (Gly), as the levels of glutamate are usually adjusted to necessity by intermediate metabolism. Cys is an amino acid abundantly present in proteins, as disulfide bridges play a fundamental role in the folding and stabilization of the tertiary structure of the proteins, thereby supporting their biological activities. Considering the major utilization of this amino acid, the competition over cysteine incorporation can affect the synthesis of many molecules; in the context of the oxidative environment created by SARS-CoV-2 infection, it is relevant to underline that, because glutathione synthesis heavily depends on cysteine availability, the levels of glutathione could be strongly curtailed by the utilization of Cys in viral proteins, and as consequence, the neutralization of free radicals would be greatly reduced.

When the balance between ROS levels and GSH buffering capacity is altered, the oxidative environment positively correlates with viral growth. Many studies report the advantage of the increased ROS production or compromised GSH synthesis exerted on viral growth. ROS generation increases significantly in response to infection with several viruses, such as Sendai virus, human respiratory syncytial virus, rhinoviruses, influenza virus, dengue virus, hepatitis C virus, and human immunodeficiency virus [24]; in SARS-CoV-2-infected patients, an increased level of two markers of oxidative stress (2-thiobarbituric acid-reacting substances (TBARS) and F2-isoprostane), as well as GSH deficiency, has recently been reported [25]. Indeed, viral infection is accompanied by the reprogramming of host cell metabolism and, above all, by the perturbation of redox metabolism; on one hand, the virus induces ROS-generating enzymes such as NADPH oxidase (NOX) and xanthine oxidase (XO), on the other hand, the antioxidant defenses are curtailed, creating unbalanced antioxidant levels [26]. For example, it has been reported that there is a decrease in GSH levels during Sendai virus replication, due to the preferential engagement of the intracellular cysteine pool in the synthesis of viral proteins [27]. The decreased GSH levels are associated with enhanced SARS-CoV-2 infection and COVID-19 severity [23,25,28]. On the contrary, as reasonably expected, glutathione inhibits the growth of viruses such as influenza, Sendai virus, and many others [29,30,31,32].

Intriguingly, ROS derived from the virus-induced pro-oxidative mechanisms favor viral replication [33,34]. The reasons explaining the advantage of increasing ROS levels can be summarized as follows.

1. Elevated ROS levels abate competition with other viral infections by modulating surface protease activity. Evidence suggests that numerous respiratory viruses exploit host proteases to enhance their spread in the host body [35] and that antioxidants serve as regulators of the protease/antiprotease balance that can prevent viral infection [36]. It has been demonstrated that some proteases used by the SARS-CoV-2 virus are redox-sensitive. Notably, ADAM17 can be directly activated by ROS and the MAP kinase family (MAPK) [37]. Once the ADAM17 is on the cell surface, it induces the shedding of diverse membrane-anchored proteins such as ACE2. The consequences of ACE2 release from the membrane are twofold: on the one hand, the soluble form of ACE2 is no longer available for viral binding, and on the other hand, the shedding leads to the downregulation of ACE2 that increases the angiotensin II levels, resulting in further rises in ADAM17 activity [38]. ROS-induced ACE2 shedding could be considered a feedback mechanism to avoid viral overload in infected cells and to spread infection in other tissues. Even if several studies have reported the beneficial and preventive role of therapeutics ACE2 in COVID-19 [39], clinical data suggest that comorbid and older patients with low membrane ACE2 and high sACE2 faced greater disease severity and fatality [40].

TMPRSS2 is also involved in other viral infections besides coronaviruses, such as Sendai virus (SeV), human metapneumovirus (HMPV), human parainfluenza viruses (HPIV), influenza A virus, and hepatitis C (HCV) [41]. Indeed, TMPRSS2 is essential for the spreading and pathogenesis of different viruses [41,42] because it is involved in proteolytic cleavage and activation of the viral surface glycoprotein hemagglutinin (HA), followed by the recognition of the host cell receptor sialic acid bound to membrane saccharides [43,44]. Oxidative stress alters both the distribution and expression of TMPRSS2, elevating its localization in the cytoplasm of intestinal epithelial cells [45] and decreasing the expression levels in the lungs [46]. Because it has been reported that the downregulation of TMPRSS2 expression could prevent the growth and spread of influenza A virus in vitro [47], it can be concluded that the decreased TMPRSS2 activity triggered by ROS could block the entry of other viruses such as influenza virus, eliminating competition and favoring SARS-CoV-2 growth in infected cells. The hypothetical advantage of ROS-mediated inhibited competition with other viral infections could be considered a negative feedback mechanism or a positive side-effect of an effective viral strategy aimed at the redox imbalance.

2. ROS generate disulfide bonds essential for viral protein stability and viral entry.

A specific balance of disulfide/thiol groups is necessary to facilitate both the viral binding to a cell and the fusion of the viral and cell membranes [48,49,50]. For example, the pro-oxidative environment created by influenza virus infection favors the folding of viral surface glycoproteins, whereas glutathione reduces disulfide bonds and hampers viral protein folding [51,52,53]. Similarly to what was reported for other viruses, recent computational work suggested that the complete reduction of disulfide bonds in ACE2 and the RBD domain of spike hampers the binding between these two proteins [49]. Accordingly, a structural and functional investigation showed that the disulfide bonds play a critical role in maintaining the proper structure of the RBD, ensuring the high-affinity interaction with ACE2 and that the reducing agents were able to inhibit viral replication [54]. Moreover, other studies demonstrated that the integrity of disulfide bonds within the RBD plays a central role in the membrane fusion step, and the reducing compounds N-acetylcysteine (NAC) and glutathione were able to inhibit viral entry [55,56].

3. ROS facilitate the replication of RNA viruses. Several studies demonstrated that the abatement of ROS prevented viral ribonucleoprotein nuclear export and viral replication in many infections. In particular, ROS can activate oxidant-sensitive p38 and NF-kB, which are two important pathways that control, for example, influenza virus replication [52,57]. In HIV-1 infection, ROS were found to stimulate viral replication through the nuclear transcription factor NF-kB [58]. In murine macrophages, the spike of SARS-coronavirus can activate NF-kB through I-kBα degradation [59], and in the human bronchial epithelial cells, viral replication of SARS-CoV-2 is associated with IKKβ upregulation [60].

This work aims to dissect which conditions favor SARS-CoV-2 entry and replication in the host cell, focusing on the relationship between viral growth and intracellular ROS levels. We propose a biochemical model that suggests the competition for Cys between viral and host proteins as a key mechanism of successful infection, and we analyze the strategy employed to select the receptor most useful for viral entry and survival. Using a novel bioinformatic procedure able to find similarities between proteins based on their amino acid content, we suggest new potential receptors for SARS-CoV-2, yet to be explored.

## 2. Materials and Methods

### 2.1. Human Protein Dataset Construction

We built a table containing all human-reviewed proteins downloaded from UniProt Knowledgebase (UniProtKB), and then we calculated their amino acid (AA) content as a relative frequency, given by the “absolute frequency” divided by the “length” of the protein.

Human protein data were obtained from the long-term preservation Database UniProtKB under the Universal Protein Resource UniProt, a comprehensive resource for protein sequence and annotation data (https://www.uniprot.org/, accessed on 10 October 2022). In our work, we considered all human-reviewed protein sequences from Swiss-Prot (https://www.uniprot.org/help/downloads, accessed on 10 October 2022) using ftp service (https://ftp.uniprot.org/pub/databases/uniprot/current_release/knowledgebase/taxonomic_divisions/, accessed on 10 October 2022) and selecting “Taxonomic division” to download the file “human_sprot_human.dat.gz”, from which we obtained the file uniprot_sprot.dat. The UniProt Knowledgebase DAT file is composed of sequence entries. Each entry corresponds to a single contiguous sequence and is composed of different types of lines, each with a format to record the different types of data which make up the entry. The line types, the line codes, and the order in which they appear in each entry are described at https://web.expasy.org/docs/userman.html#genstruc (accessed on 10 October 2022). We adopted the nomenclature and symbolism from IUPAC-IUC Joint Commission on Biochemical Nomenclature (JCBN) for amino acids and peptides (https://febs.onlinelibrary.wiley.com/doi/10.1111/j.1432-1033.1984.tb07877.x, accessed on 10 October 2022). Proteins were labeled according to UniProtKB nomenclature. The total number of human-reviewed proteins downloaded was 20,398 on 10 October 2022.

### 2.2. Viral Protein Dataset Construction

The sequence of each viral protein of interest was downloaded, and then their AA content was calculated as a relative frequency.

The consensus sequences of the SARS-CoV-2 circulating variants were obtained from ViralZone (https://viralzone.expasy.org/9556, accessed on 10 October 2022), which contains the link to the “Spike protein UniProt” and/or “Spike protein NCBI”. For our analysis, we used spike protein of the Variants of Concern (VOC): Alpha B.1.1.7, Beta B.1.351, Gamma P1, Delta B.1.617.2, Omicron BA.1, Omicron BA.2, Omicron BA.2.12.1, Omicron BA.2.75, Omicron BA.4, Omicron BA.5. The sequences of the polyproteins R1AB “P0DTD1 1AB_SARS2” and R1A “P0DTC1 R1A_SARS2”, and the structural proteins E “P0DTC4 VEMP_SARS2”, M “P0DTC5 VME1_SARS2”, and N “P0DTC9 NCAP_SARS2” for severe acute respiratory syndrome coronavirus 2 (2019-nCoV) (SARS-CoV-2) were obtained from https://COVID-19.uniprot.org/uniprotkb (accessed on 10 October 2022). The consensus sequences of other viral proteins were downloaded from UniProtKB. In detail, we analyzed the following proteins: the major surface glycoprotein G of human respiratory syncytial virus A “P03423 GLYC_HRSVA”, the genome polyprotein of hepatitis C virus genotype 1b “P26662 POLG_HCVJA”, the fusion glycoprotein F0 of human respiratory syncytial virus A “P03420 FUS_HRSVA”, the hemagglutinin of influenza A virus “P03454 HEMA_I33A0”, and the envelope glycoprotein gp160 of HIV-1 “C7TQ85 C7TQ85_9HIV1”.

### 2.3. Construction of Protein Datasets with Amino Acid Relative Content

The final dataset contained different tables, each with all the human-reviewed protein data and one viral protein queued as the last row. In all tables, the AA content was calculated as a relative frequency and was used to measure the similarity between human and viral proteins.

The Uniprot DAT file “uniprot_sprot_human_10102022.dat” from UniProtKB/Swiss-Prot was parsed through a Perl script written specifically to extract the amino acid contents for each human protein using the sequence line which contains the amino acids and the length of the sequence.

For each human protein, we saved the ID, the length, and the relative frequency for each amino acid type in the sequence, using the one-letter nomenclature and symbolism from IUPAC-IUC Joint Commission on Biochemical Nomenclature (JCBN). The relative frequency was a five-digit floating-point number with three digits after the decimal point, and it was calculated by dividing the absolute frequency by the length of the sequence.

For each SARS-CoV-2 variant from ViralZone, we used the ProtParam (ExPASy—ProtParam tool) to compute the protein parameters and in particular the AA composition.

We decided to focus on the spike from Omicron BA.1 for further analysis because spike proteins produced by further variants of Omicron were very similar in amino acid content to each other.

Starting from the dataset of human-reviewed proteins and the spike from Omicron BA.1 amino acids contents, we created new tables with the OMICRON BA.1 AA data queued to human data, saved with and without cysteine amino acid content in xlsx and csv format. The same procedure was followed for all viral proteins.

### 2.4. Evaluation of Protein Similarities by Hierarchical Cluster Protein Analysis

We decided to exploit the clustering analysis for evaluating the protein similarities. Cluster analysis was applied to the built datasets; first, we searched for the methods that best conformed to the data analysis, and then we measured the similarity between human and viral proteins. Similarities were calculated by the “average” method and then validated by at least 3 out of the 4 best methods.

Specifically, a hierarchical cluster protein analysis was performed on the OMICRON BA.1 AA data queued to the human data table exploiting the Fastcluster R package [61]. We selected the hierarchical clustering algorithm given that (i) it is not necessary to know a priori the number of clusters, and (ii) its result is more interpretable and informative in terms of similarity among the input elements (in our case, proteins).

Hierarchical cluster analysis was performed using the seven most widely used methods offered by the package: “single”, “complete”, “average”, “mcquitty”, “ward.D”, “ward.D2”, “centroid”, or “median”. These methods differ with respect to how the proximity between any two clusters is defined. Therefore, the hierarchical algorithm starts by treating each protein as a singleton cluster. Then, it continuously merges pairs of clusters with lower proximity until all clusters have been merged into one big cluster containing all objects. Thus, the height of the merging (known as cophenetic distance) indicates the (dis)similarity between two clusters. Let us note that the higher the height of the merging, the less similar the objects are. The result is a tree-based representation of the objects, named a dendrogram.

For each algorithm, we calculated the cophenetic correlation coefficient [62], which measures how faithfully a dendrogram preserves the pairwise distances between the original data points. The coefficient values vary between 0 (low-quality solution) and 1 (high-quality solution). In this sense, we were able to filter the methods with low performance.

The hierarchical cluster analysis was employed to search for similarity between proteins based on their amino acid content, considering either the whole amino acid content (with Cys) or excluding cysteine content from the analysis (without Cys). The analysis was carried out following two strategies:

1. We used the method with a higher cophenetic correlation coefficient (“average”) to measure the similarities between human proteins and viral variants in terms of cophenetic distance. The heights of dendrograms obtained from the hierarchical clustering analysis were normalized to the maximum height to make comparison among the different analyses possible.
2. We chose the four best methods (“average”, “single”, “median”, and “centroid”) with a cophenetic correlation coefficient greater than 0.7, and for each method, we selected a subset of protein clusters such that it belongs to the 5% of proteins closest (depending on the cophenetic distance) to spike from Omicron_BA.1. The resulting four subsets were merged, obtaining a list of proteins, with the proteins most similar to spike as calculated by at least 3 out of the 4 best methods. This approach was used to validate the similarity of proteins of interest.

## 3. Results

### 3.1. A Biochemical Model Envisaging the Competition for Cysteine as a Key Mechanism Promoting the Infection and the Selection of Host Receptors

**Figure 1:**
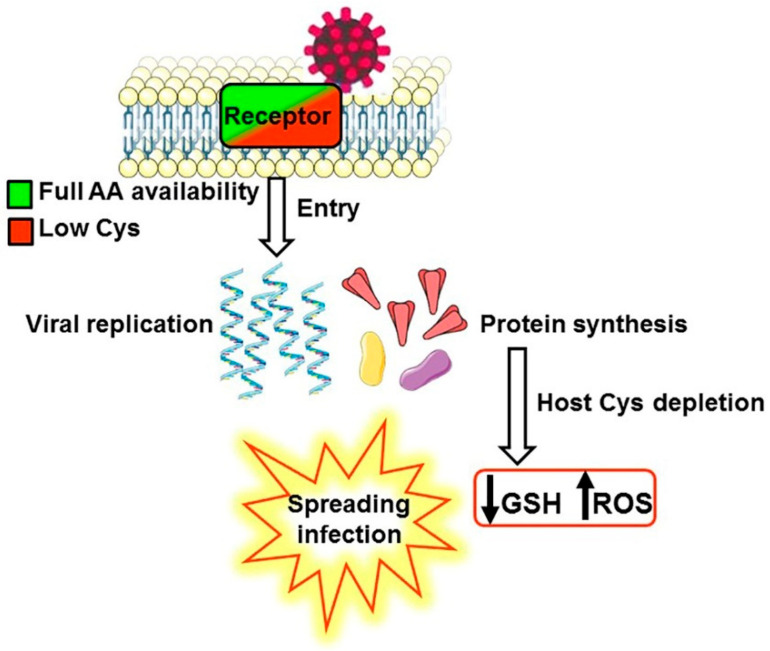
Schematic representation of the biochemical model proposed to describe how the binding to a receptor poor of Cys can drive the viral entry into a cellular milieu suitable for viral replication.

We carried out a biochemical analysis that could reveal the intracellular optimal conditions for viral infection. Because mRNAs have a short half-life (median of 7 h) [63], protein synthesis occurs only when the availability of amino acids is sufficient to maintain a rapid translation appropriate to the lability of the mRNA [64]. Therefore, protein synthesis depends on mRNA production and the local availability of amino acids. When the latter becomes limiting, the proteins exploiting the available amino acids are preferentially produced [65]. SARS-CoV-2 competes with the host for protein synthesis; indeed the virus impairs cellular translation [66] to incorporate the amino acids in its proteins. If a limiting Cys pool is depleted by intense viral replication, the synthesis of GSH is reduced, the oxidative stress is enhanced, and viral infection can succeed. Intracellular limiting availability of some amino acids would select the synthesis of some proteins, both soluble and membrane-bound, built with the available amino acids. We hypothesize that when a limiting pool of Cys amplify the synthesis preferentially towards the production of plasma membrane proteins particularly poor of this amino acid but rich of all amino acids necessary for viral proteins, the viral binding to these receptors will ensure the entry into a cell that possesses the conditions suitable for viral replication. This biochemical model lays the foundations for a similarity search between viral and human proteins, leading to the discovery of new receptors of SARS-CoV-2. The biochemical model is depicted in Figure 1.

### 3.2. The Selection of ACE2 as the Main SARS-CoV-2 Receptor Based on Its Amino Acid Composition

Our biochemical model predicts that viral infection is favored by intracellular conditions that comply with its survival needs: the establishment of a pro-oxidative environment by stimulation of ROS production and the full availability of all amino acids necessary to produce massive amounts of viral proteins. We set out to analyze the composition of ACE2, taking into account these necessary conditions.

#### 3.2.1. Cysteine Content of Viral Proteins and ACE2

Since the increased ratio ROS/GSH supports viral growth, the best strategy for virus survival would be the enhancement of ROS production and the fall of glutathione synthesis. The latter is strictly dependent on cysteine availability, which could be undermined by an intense production of viral proteins rich in cysteine. Indeed, this amino acid is abundantly present both in R1A and R1AB polyproteins, which are immediately produced upon viral entry, and in all variants of the spike, which contain 3.1% Cys, more than the average of human proteins (median 2.1%) and much more than ACE2, limited to 1% total amino acid content (Table 1). Notably, Cys content remains unaltered in all spike variants, as shown in Table 1. The envelope protein (E) even reaches 4% Cys, whereas the M protein is quite close to the average content of human proteins. Taken together, these results reveal that most SARS-CoV-2 proteins have a high content of Cys, or at least not as low as ACE2, except for N, supporting the reasonable hypothesis that the outburst of viral replication could deplete the cysteine pool and prevent the adequate synthesis of glutathione.

#### 3.2.2. Composition of Viral Proteins and ACE2

As we demonstrated in our previous works [67,68], the protein composition is a decisive factor in the protein synthesis rate due to the variable intracellular levels of each amino acid, which depend on the balance between uptake, biosynthesis, and competitive utilization. This principle can be applied to viral protein synthesis too; therefore, the production of polyproteins first and essential viral proteins next would benefit from the adequate availability of some limiting amino acids. It is reasonable to assume that one relevant contribution to selective transmission advantage would be the recognition of surface proteins mirroring the intracellular optimal growth conditions and the exploitation of such proteins for viral entry. Based on this possibility, the optimal receptor would have an amino acid composition very similar to viral polyproteins or spike except for lower Cys, so that SARS-CoV-2 could synthesize its proteins, consume the limited host cysteine pool, trigger oxidative stress, and facilitate replication. To validate our hypothesis, we evaluated the amino acid composition of ACE2 and viral proteins (spike variants and polyproteins), and we found a striking similarity between them, as shown in Table 2. The similarity between ACE2 and each viral protein was derived from the hierarchical cluster protein analysis using the “average” algorithm, as described in Methods. The high similarity was found both when considering the content of all amino acids except Cys, and when comparing the whole set of amino acids; in fact, the high resemblance in amino acid content makes the difference between analysis with and without Cys negligible. To reinforce the strength of our analysis, we discovered that the similarity to ACE2 is much lower for proteins produced by other viruses not infecting the host cells through ACE2. In Table 2, we show the increased distance from ACE2 relative to the glycoprotein G of human respiratory syncytial virus A (HRSVA) and the polyprotein of hepatitis C virus (HCV). The relevance of the similarities found in this analysis can be better appreciated if compared to the distribution of similarity values in the whole datasets of human proteins, as shown in Supplementary Figure S1.

The strong similarity revealed by this analysis, together with the observation that ACE2 shows a smaller content of Cys compared to viral proteins (1% versus 3% respectively, as shown in Table 1), can explain the selection of ACE2 as the preferred receptor for SARS-CoV-2 entry and support a reasonable explanation for the consequent oxidative burst, based on our biochemical model.

### 3.3. The Search for Novel Putative Receptors or Co-Receptors for SARS-CoV-2

**Figure 2:**
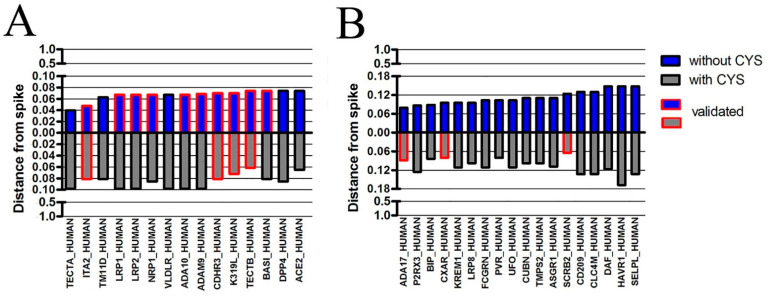
Similarities with spike of 32 proteins of interest. The similarity is expressed as the distance from spike, by a scale of values ranging from 1 (no similarity) to 0 (identical proteins). (**A**) A total of 14 proteins were more similar to spike than ACE2 in the analysis without Cys. (**B**) A total of 18 proteins were close but slightly less similar to spike than ACE2 in the analysis without Cys. Many similarities were confirmed by four algorithms (red-outlined bars—validated).

Based on conclusions reached by the analysis of ACE2 features, we wondered whether the same approach could be exploited to search for novel receptors for SARS-CoV-2, which would be surface proteins very similar to spike but with a lower content of Cys. Since the most recent variant, Omicron, shows a more general tropism than the Alpha variant, we hypothesized that many different receptors or co-receptors, unknown or yet to be verified, could mediate viral entry in different organs. The search was developed in three steps:

1. We calculated the relative content of each amino acid except Cys in all human proteins, and we searched the proteins most resembling the spike of the Omicron variant. We decided to exclude Cys content because the potential receptors should be almost identical to spike except for Cys. The similarity was obtained from the hierarchical cluster protein analysis with the “average” method as described in Methods. We found 1648 human proteins with a similarity to spike higher than or equal to the value found for ACE2. Among the listed proteins, 14 potential receptors have caught our attention and their similarity with spike is shown in Figure 2A.
2. We searched the scientific literature and identified 18 surface proteins related to viral infection (SARS-CoV or other viruses); we calculated their similarity and discovered that they were very close to spike, as shown in Figure 2B. The total 32 proteins of interest (14 from step 1 and 18 from step 2) are listed in Supplementary Table S1.
3. We validated the similarities using four methods. In this procedure, as described in Methods, a subset of proteins shared among the four methods with good cophenetic correlation coefficient was obtained, containing the proteins most similar to the spike of Omicron_BA.1. We discovered that 10 proteins out of 32 were consistently very similar to spike, as indicated in Figure 2A,B by red-outlined bars.

Steps 1–3 were repeated considering in addition the content of Cys in human proteins. We obtained 261 human proteins with a similarity to spike higher than or equal to the value found for ACE2. Among these, only one protein was interesting relative to virus infection (TECTB). Then, we calculated the similarity to spike for proteins described in steps 1–2 but considering the content of Cys too. A total of 7 proteins out of 32 were consistently very similar to spike when their similarities were validated by the four methods. These results are shown in Figure 2.

Finally, the 32 proteins of interest were evaluated in terms of relative Cys content and compared with the spike of the Omicron variant. Considering that spike has a relative Cys content equal to 0.03 (3%), we discovered that several proteins had less Cys than spike, and we hypothesize that these proteins of interest could be receptors or co-receptors which during SARS-CoV-2 infection would signal intracellular shortage of Cys, predicting a burst of oxidative stress when used as viral entry mediators. The results of the Cys analysis are shown in Table 3, in which the receptors with a low content of Cys and their tissue localization are highlighted in red.

## 4. Discussion

We believe that viral infection can be favored when intracellular conditions comply with two requirements essential for replication: first, the full availability of all amino acids necessary to produce massive amounts of viral proteins; second, the establishment of an oxidative environment, which is created on one hand by stimulation of ROS production and on the other hand by competitive incorporation of Cys into viral proteins, with the consequence of depleting GSH stores. Both mechanisms contribute to redox imbalance; the former is mediated by many pro-oxidant pathways such as the activation of NADPH oxidase [69] and xanthine oxidase [70] and the induction of mitochondrial damage due to ROS release [71] and to the impairment of the mitochondrial redox system triggered by nsp10 [72]. To curb glutathione synthesis, Cys must be heavily consumed by other metabolic pathways such as protein synthesis. Cys is particularly abundant in the SARS-CoV-2 protein set. Among all proteins produced by viruses that infect humans, the SARS-CoV-2 spike features the highest cysteine content, especially in the cytoplasmic domain, which is essential for palmitoylation and membrane fusion [73]. The intramolecular disulfide bridge of the spike protein between Cys-488 and Cys-480 in its RBD is important for the molecular structure requested for ACE2 binding. The thiol-reducing agents NAC and GSH hamper the binding and infectious activity of spike [56]. In our novel analysis, the polyprotein source of all non-structural viral proteins shows a Cys content similar to spike (3%), and Cys is even more abundant in the envelope protein E (4%). Furthermore, it has been reported that two more proteins of SARS-CoV-2 are unusually rich in Cys residues, namely the immune modulatory accessory protein ORF8 [74] and the main protease (M^pro^), which is a 3-chymotrypsin-like protease with a critical role in the production of viral proteins [75]. Remarkably, the M^pro^ Cys content (3.92% of residues) is even higher than the Cys content of spike and nearly double the average content of human proteins (median value of 2.1%). Besides the catalytic Cys, several additional surface Cys residues of M^pro^ are not involved in disulfide bonds and probably protect the active-site Cys145 from ROS-triggered oxidative damage [76]. Overall, data from previous works and our analysis reveal that SARS-CoV-2 incorporates a high amount of Cys in its proteins; it is reasonable to conclude that SARS-CoV-2 is highly competitive over the Cys intracellular pool, and this competition can lead to GSH depletion. In agreement with this hypothesis, we have calculated that some proteins of other viruses creating redox imbalance have the same Cys enrichment, for example, the genome polyprotein of Hepatitis C virus (3.3%), the fusion glycoprotein F0 of Human respiratory syncytial virus A (2.8%), the hemagglutinin of Influenza A virus (2.7%), and the envelope glycoprotein gp160 of HIV (2.6%).

We show that ACE2 is an optimal receptor for SARS-CoV-2 in terms of amino acid composition due to the high similarity in all amino acid content except Cys. The latter is much lower than what is utilized in viral proteins, and it is also lower than the average content of Cys of all human proteins. Based on our biochemical model, the presence of ACE2 on membranes can be interpreted as a marker of intracellular low availability of Cys, which is still sufficient for ACE2 synthesis and favors the production of this protein over others. If we consider that, intracellular availability of amino acids may be a limiting factor in protein biosynthesis. Even if we propose ACE2 as a qualitative avatar of cytoplasm amino acid composition, we cannot rule out the opposite causality direction; in other words, the receptor could be selected because, in the first phases of viral replication, it is not affected by the progressive shortage of Cys. The composition of ACE2 ensures that viral entry will be followed by strong competition over the limited pool of Cys between host glutathione production and viral protein factory. The latter will prevail, unleashing the mechanisms of ROS production and depleting the main antioxidant defense, which will not be replenished due to the shortage of Cys. Considering the broad spreading of virions in the whole organism, the virus reproduces best in tissues where ACE2 is most abundant, and these tissues are the most prone to oxidative stress and damage.

It is reasonable to hypothesize that the viruses described by our analysis as potent Cys consumers should deplete intracellular Cys content and abate GSH levels, making the host cell more prone to oxidative stress and a better target for SARS-CoV-2 infection. Indeed, we were very excited to discover that HCV, influenza virus, and HIV infection induce the expression of ACE2 (poor of Cys) and facilitate SARS-CoV-2 entry and replication [77,78,79]. We believe that these experimental pieces of evidence confirm the soundness of our biochemical model.

This study assumes that the amino acid composition of proteins reflects the availability of amino acids imposed by the cellular context. The validity of this concept has been demonstrated in our previous works [67,68,80]. For example, we have shown that the oxygenated environment is advantageous to the biosynthesis of proteins enriched in glutamate (Glu). At the same time, the hypoxic condition increases the availability of glutamine (Gln) and thus favors the translation of glutamine-rich proteins. We demonstrated that, in the epidermis model, the ratio Glu/Gln of the epidermal proteins was directly proportional to the oxygenation of the layer expressing the protein, evidently due to the conversion of glutamate to glutamine driven by the hypoxic context. Moreover, the analysis of two chromosome loci suggested that gene clustering may represent an adaptation for responding to amino acid availability [67]. The present study suggests that cysteine is another critical amino acid whose availability can drive protein expression. Levels of Cys may become limited due to its extensive utilization. Indeed, cysteine is broadly employed as an antioxidant, in enzyme catalysis (Cys-dependent proteases or iron–sulfur clusters of many enzymes), and the construction of small peptides such as glutathione. In all proteins, its content is critical and necessary to build disulfide bridges and for this reason is not replaceable without loss of structure. When the levels of this critical residue become limiting, the competition between biosynthetic pathways favors the most substantial pathway. This is what occurs in the fight between viral growth and host defenses.

The natural evolution of the SARS-CoV-2 pandemic has generated many viral variants as a mechanism of viral adaptation to the host. It is interesting to note that all variants have the same content of Cys in their spike protein, as proof of how essential this amino acid is in the structure and function of spike. With the appearance of new variants, the tropism of SARS-CoV-2 has changed too, reinforcing the possibility that the entry of the virus depends on receptors other than ACE2, and it can be concluded that the binding to novel receptors has become crucial for the evolving viral infection. Indeed, an extraordinary feature of coronaviruses is the diversity of receptor usage; some of them have been described, and the binding tested in specific models, but many remain to be discovered. In this study, we applied the principles of our biochemical analysis of ACE2 to all human proteins, and using bioinformatic and mathematical tools, we matched the amino acid content of human proteins with the composition of spike produced by the latest variant, Omicron BA.1. We found 32 human proteins very close to the spike in terms of amino acid content, both including and excluding Cys in this analysis of similarity. Most of these potential SARS-CoV-2 receptors have less Cys than spike, and we hypothesize that these proteins of interest signal an intracellular shortage of Cys, predicting a burst of oxidative stress when used as viral entry mediators. Overall, it can be assumed that natural selection drove spike evolution to recognize membrane hallmarks (including ACE2) of compliant translation machinery in the cytoplasm.

Remarkably, many putative or confirmed receptors previously described by others are present in our list, to support the validity of our biochemical approach. The following proteins were described in previous works as cellular targets of SARS-CoV-2: GRP78, DPP4, UFO, Basigin/CD147, NRP1, the lectins CD209 and CD209L/CLC4M [12], ASGPR1 [81]. GRP78 (also known as HSPA5 or BiP) is broadly expressed in the respiratory mucosa [82] and is a potential host-cell receptor for SARS-CoV-2 [83]. Its mRNA levels are much higher than those of ACE2 in the lungs, indicating that GRP78 should play an important role in SARS-CoV-2 entry through the lungs [84]. Very recently, Shin et al. observed an upregulation of GRP78 in SARS-CoV-2-infected cells [85], whereas Shaban et al.’s recent report suggested that elevated GRP78 levels may contribute to some of the antiviral effects of the ER stress inducer thapsigargin [86]. From these studies, it can be concluded that the pharmacological manipulation of GRP78 warrants further experimental and pre-clinical work, promising potentially beneficial therapeutic effects in COVID-19. CD209L and CD209 are members of the C-type lectin superfamily and are described as mediators of viral pathogenesis. The receptor-binding domain (RBD) of the SARS-CoV-2 S protein binds to CD209L and CD209, mediating SARS-CoV-2 entry. CD209L is expressed in human endothelial cells and mediates cell adhesion and the formation of capillary tubes, whereas CD209 is primarily expressed in dendritic cells and tissue-resident macrophages [18]. The asialoglycoprotein receptor 1 (ASGPR1) is a high-capacity C-type lectin receptor mainly expressed in mammalian hepatic cells. Studies in vitro show that spike protein interacts with the ASGR1 in human hepatocytes [81].

It is exciting that, in our analysis, several members of the “A Disintegrin And Metalloprotease” (ADAM) family of sheddases were found to be very similar to spike (Omicron variant) in terms of amino acid composition, as well as the spike-activating proteases TMPRSS2 and TMPRSS11D. ADAM17 and ADAM10 have a role in ACE2 shedding; in addition, they can cleave the SARS-CoV-2 spike protein in vitro, indicating that they could contribute to the priming of spike [87]. Moreover, it has been reported that ADAM9 affects viral uptake or replication in vitro since ADAM9 inhibition decreases SARS-CoV-2 infection [88]. Based on our biochemical considerations on amino acid availability, we conclude that, in addition to receptors, the composition of the proteases facilitating SARS-CoV-2 entry must guarantee an intracellular milieu fit for viral replication.

Another exciting discovery is that many proteins which are known as receptors for other viruses have been discovered in this study as very similar to spike: SCARB2/LIMP-2, CDHR3, CAR, LRP-8/ApoER2, LRP-1, VLDLR, ITA-2, DAF, PSGL1, Kremen-1, PVR/CD155, FcRn, HAVR1/KIM-1, and KIAA0319L/AAVR. Their exploitation for entry could be advantageous to SARS-CoV-2 infection, hampering the uptake of other viral entities which could compete for host substrates.

Finally, some proteins not yet identified as viral binding partners have caught our attention because they could be responsible for the broad tissue tropism displayed by the Omicron variant. TECTA and TECTB are major components of the tectorial membrane of the inner ear involved in sound transduction; mutations of these membrane proteins are associated with hearing abnormalities. P2RX3 is required for the perception of pain and taste in the cells of the gustatory system; Megalin and Cubilin are highly expressed in kidney, where they are active as multiligand endocytic receptors. Among their substrates, renal uptake of 25-hydroxyvitamin D3 could be affected by the alteration of Megalin and Cubilin membrane turnover elicited by SARS-CoV-2 binding and entry. Vitamin D deficiency has been associated with COVID-19 severity and outcome; therefore, the possible involvement of Megalin and Cubilin deserves further investigation.

In addition to the well-characterized pulmonary manifestations, SARS-CoV-2 induces a broad range of clinical abnormalities, including neurologic, ocular, cardiac, gastrointestinal/hepatic, renal, and hematologic alterations, and also hearing and taste impairment; the increased thrombotic risk is possibly related to direct viral infection of the endothelium [89]. These diverse manifestations may be related to viral tropism and host immune responses. The analysis presented in this study can boost the investigation of novel receptors responsible for viral tropism and can contribute to explaining the pro-inflammatory and pro-oxidative consequences of viral infection in the target tissues.

## 5. Conclusions

In this study, we propose a biochemical model illustrating how the competition for Cys can lead to the oxidative stress supporting viral growth; based on these considerations, we define the characteristics shared by receptors or co-receptors suitable to promote SARS-CoV-2 infection, and we present a list of potential candidates, which was obtained using bioinformatic analysis of the amino acid composition of human proteins followed by a similarity search.

## Figures and Tables

**Table 1:** Relative Cys content of SARS-CoV-2 proteins (spike variants, polyproteins, and structural proteins SP), human ACE2, and average content of all human proteins (median and quartiles).

Spike:	Alpha	Beta	Delta	Gamma	BA.1	BA.2	BA.2.12.1	BA.2.75	BA.4	BA.5
	0.031	0.031	0.031	0.031	0.031	0.031	0.031	0.031	0.032	0.032
Polyproteins:	R1A	R1AB								
	0.031	0.032								
SP:	E	M	N							
	0.040	0.018	0							
Human proteins:	ACE2	Median of all proteins (Q1–Q3)								
	0.010	0.021 (0.01–0.03)								

**Table 2:** The similarity between ACE2 and SARS-CoV-2 proteins (spike variants and polyproteins R1AB and R1A) was measured by the “average” method, based on whole amino acid content except Cys (without Cys) or based on complete amino acid content (with Cys). The scale of values goes from 1 (no similarity) to 0 (identical proteins). As a negative control, proteins from viruses that do not use ACE2 as a receptor were analyzed (glycoprotein G of human respiratory syncytial virus A and genome polyprotein of hepatitis C virus). Blue represents ACE2 in the analysis without Cys. Grey represents ACE2 in the analysis with Cys.

	Alpha	Beta	Delta	Gamma	BA.1 ^a^	GLYC		POLG		
						RIAB	R1A	HRSVA	HCV	
ACE2	0.074	0.074	0.074	0.074	0.074	0.071	0.070	0.219	0.098	without Cys
ACE2	0.082	0.082	0.082	0.082	0.065	0.072	0.070	0.235	0.092	with Cys

^a^ Omicron BA.1.

**Table 3:** Proteins of interest were analyzed for their relative Cys content. Tissues highlighted in red are proposed as most prone to oxidative stress because they are poor in cysteine.

Main Target Tissue	SARS-CoV-2 Receptor (Putative or Verified)	Relative Cys Content	Signal for Oxidative Stress Relative to Spike (Cys Less than 0.031)
**Ubiquitous**	ACE2	0.010	**YES**
	LRP1	0.073	NO
	TMPRSS2	0.045	NO
	TMPRSS11D	0.022	**YES**
	ADAM17	0.042	NO
	ADAM10	0.048	NO
	ADAM9	0.054	NO
	DPP4	0.016	**YES**
	UFO	0.025	**YES**
	CD209/DC-SIGN	0.022	**YES**
**Ear**	TECTA	0.068	NO
	TECTB	0.040	NO
**Taste**	P2RX3	0.030	**YES**
**Nasal airway epithelium**	CDHR3	0.008	**YES**
	NRP1	0.024	**YES**
**Lungs**	BiP/GRP78	0.003	**YES**
	CDHR3	0.008	**YES**
**Heart**	CAR	0.027	**YES**
	Basigin	0.018	**YES**
**Intestine**	SCARB2	0.017	**YES**
	CAR	0.027	**YES**
	ITA-2	0.019	**YES**
	DAF	0.047	NO
	PSGL-1	0.007	**YES**
	Kremen-1	0.036	NO
	PVR	0.022	**YES**
	FcRn	0.016	**YES**
**Kidney**	LRP2/megalin	0.071	NO
	Cubilin	0.043	NO
	HAVR1	0.019	**YES**
**Nervous system**	LRP-8	0.065	NO
	VLDLR	0.077	NO
	K319L	0.018	**YES**
	PVR	0.022	**YES**
	SCARB2	0.017	**YES**
**Liver**	ASGR1	0.034	NO
	VLDLR	0.077	NO
**Endothelium**	CD209L/L-SIGN	0.023	**YES**
**Platelets**	LRP-8	0.065	NO

## Data Availability

The data presented in this study are available within the article.

## Supplementary Materials

The following supporting information can be downloaded at: https://www.mdpi.com/article/10.3390/antiox12020483/s1, Figure S1: Similarity of viral proteins to ACE2 compared to the distribution of similarity values in the whole datasets of human proteins. Table S1: Proteins of interest. References [90,91,92,93,94,95,96,97,98,99,100,101,102,103,104,105,106,107,108,109,110,111,112,113,114,115,116,117,118,119,120,121,122,123,124,125,126,127,128,129,130,131,132,133,134,135,136,137,138,139,140,141,142,143,144] are cited in the Table S1.
