# Supplementary figures and images for: How the Competition for Cysteine May Promote Infection of SARS-CoV-2 by Triggering Oxidative Stress
Source: Antioxidants (Basel). 2023 Feb 14;12(2):483. doi: 10.3390/antiox12020483 (PMC9952211; doi:10.3390/antiox12020483)

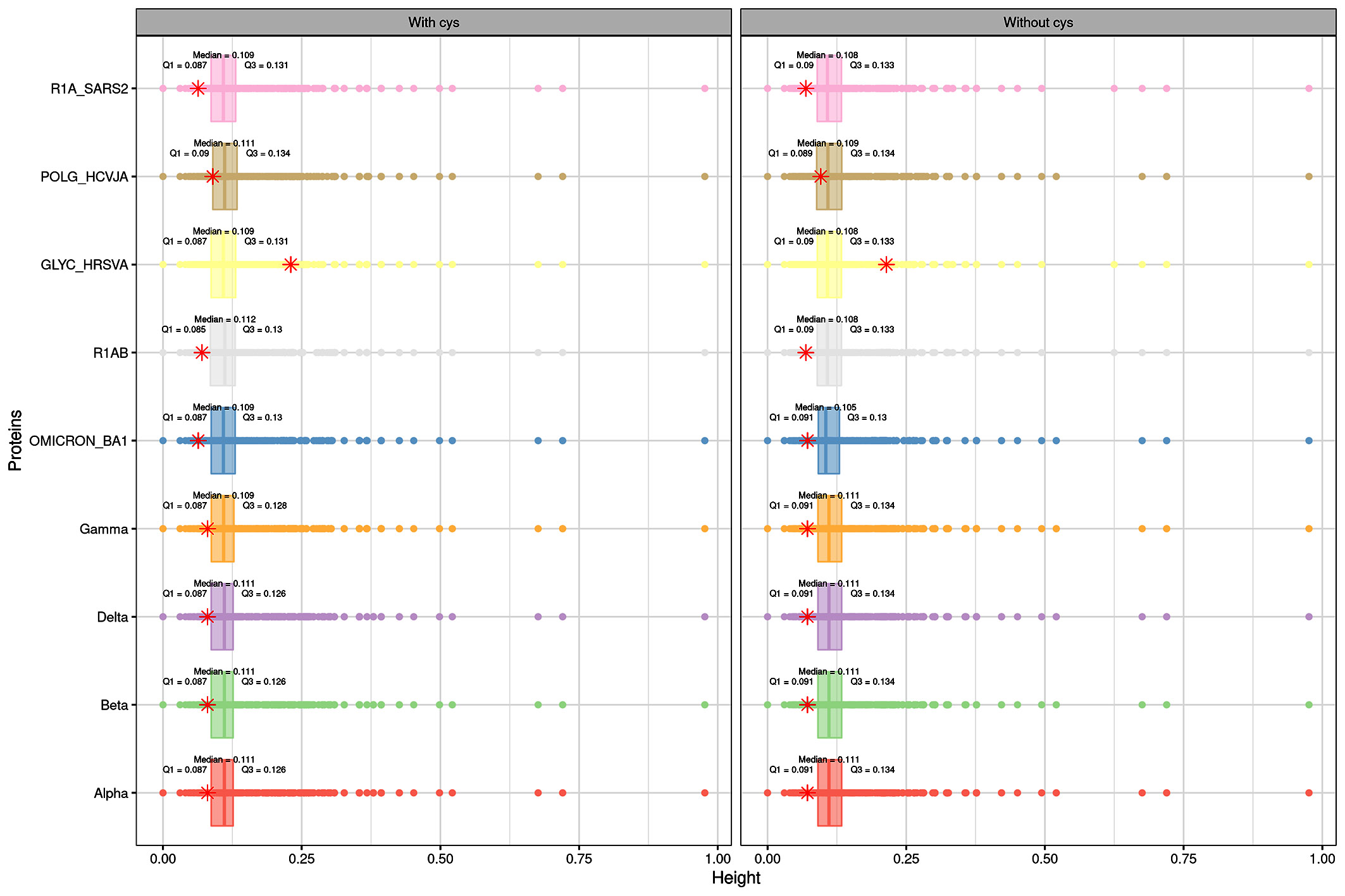

Supplement: Supplementary file 1 [file antioxidants-12-00483-s001.zip › Figure S1.jpg]
